# The application of traditional machine learning and deep learning techniques in mammography: a review

**DOI:** 10.3389/fonc.2023.1213045

**Published:** 2023-08-11

**Authors:** Ying’e Gao, Jingjing Lin, Yuzhuo Zhou, Rongjin Lin

**Affiliations:** ^1^ School of Nursing Fujian Medical University, Fuzhou, China; ^2^ Department of Surgery, Hannover Medical School, Hannover, Germany; ^3^ Department of Nursing, the First Affiliated Hospital of Fujian Medical University, Fuzhou, China

**Keywords:** breast cancer, machine learning, mammogram image, deep learning, diagnose

## Abstract

Breast cancer, the most prevalent malignant tumor among women, poses a significant threat to patients’ physical and mental well-being. Recent advances in early screening technology have facilitated the early detection of an increasing number of breast cancers, resulting in a substantial improvement in patients’ overall survival rates. The primary techniques used for early breast cancer diagnosis include mammography, breast ultrasound, breast MRI, and pathological examination. However, the clinical interpretation and analysis of the images produced by these technologies often involve significant labor costs and rely heavily on the expertise of clinicians, leading to inherent deviations. Consequently, artificial intelligence(AI) has emerged as a valuable technology in breast cancer diagnosis. Artificial intelligence includes Machine Learning(ML) and Deep Learning(DL). By simulating human behavior to learn from and process data, ML and DL aid in lesion localization reduce misdiagnosis rates, and improve accuracy. This narrative review provides a comprehensive review of the current research status of mammography using traditional ML and DL algorithms. It particularly highlights the latest advancements in DL methods for mammogram image analysis and offers insights into future development directions.

## 1 Introduction

Golobocan’s latest cancer report (1) states that in 2020, approximately 19.3 million newly diagnosed cancer cases were reported worldwide, resulting in 10 million cancer-related deaths. Among these cases, female breast cancer accounted for about 2.3 million new cases, representing 11.7% of all cancer cases and surpassing lung cancer as the most prevalent malignant tumor. Breast cancer has emerged as a leading malignancy among women, significantly endangering their physical and mental well-being. The mortality rate of breast cancer patients has been decreasing due to advancements in medical technology and early detection. From 1975 to 1989, breast cancer mortality experienced a yearly increase of 0.4 percent but has steadily declined since reaching its peak in 1989. Between 1989 and 2020, there was a remarkable 43 percent decrease in mortality, resulting in a reduction of 460,000 breast cancer-related deaths (2). The decrease in breast cancer mortality can be attributed to improved and more targeted treatment methods, as well as early screening practices. Consequently, early diagnosis and treatment play pivotal roles in enhancing the prognosis of these patients.

Currently, the primary techniques used for the early diagnosis of breast cancer include mammography, magnetic resonance imaging (MRI), ultrasonography (US), computed tomography (CT), and pathological examination (3). Mammograms, among these methods, are relatively inexpensive, straightforward, and rapid. They enable the detection of even minor breast changes that may go unnoticed during manual examination, thereby enhancing diagnostic accuracy (4). Hence, despite the emergence of new technologies for breast cancer diagnosis, mammography remains the simplest and most frequently employed tool for early breast cancer screening (5). However, following mammography imaging, the clinical interpretation and analysis of images necessitate significant time and labor costs, relying heavily on the expertise of clinicians for film interpretation. Diagnosis by doctors is subjective, and their individual experience levels vary. Even experts find it challenging to make immediate and accurate judgments when faced with diverse breast abnormalities, leading to the potential for missed diagnoses and misdiagnoses. Furthermore, training experienced clinicians requires substantial investments of time and effort, posing challenges for professions facing a shortage of technical expertise. Therefore, the development of new technologies is necessary to address these aforementioned challenges. The advancement and widespread adoption of computer technology have facilitated the availability of sufficient computational power for medical image analysis and processing. This, in turn, mitigates the reliance on the expertise level of doctors to some extent, with ML methods proving particularly suitable under these circumstances.

**Figure 1:**
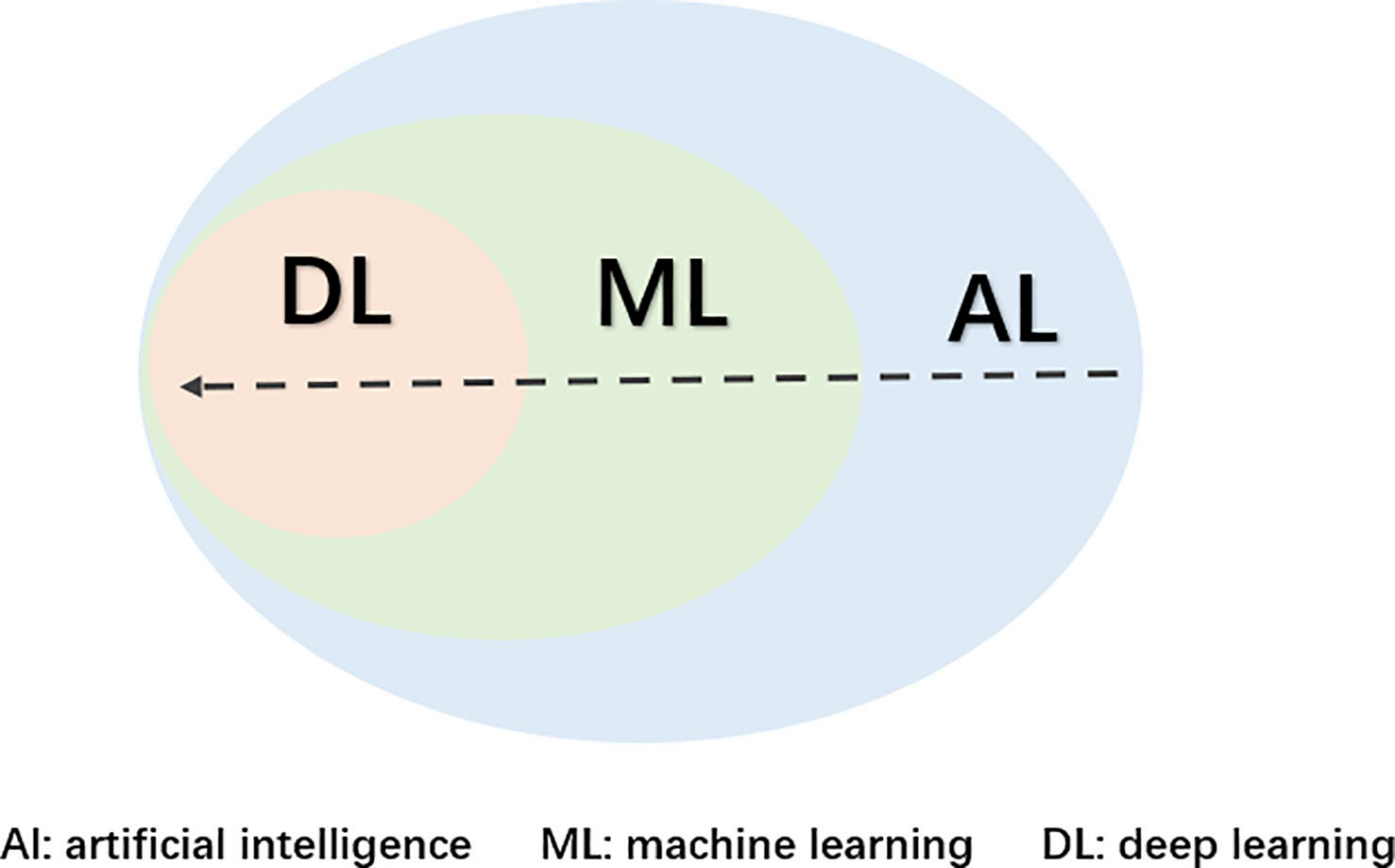
Relation between AI、ML and DL.

ML is a subfield of artificial intelligence (AI) that emerged in the 1980s (**Figure 1**) (6). By training and learning, the machine extracts the most relevant features from the dataset and constructs a model to handle unfamiliar data (7). ML comprises three main categories: supervised learning, unsupervised learning, and reinforcement learning. In supervised learning, well-labeled data is used to train a model, which then generates predictions or classification outcomes based on the provided data (8); In unsupervised learning, computers address pattern recognition problems using unlabeled training samples. For instance, unsupervised DL algorithms can identify distinguishing features between benign and malignant nodules, classifying them into respective categories (8); Conversely, reinforcement learning relies on reward feedback to maximize returns or accomplish specific objectives (9). The most frequently employed supervised learning methods include Artificial Neural Networks (ANN), Support Vector Machine (SVM) (10), and Random Forest (RF) (11, 12). Clustering algorithms, such as K-Means (13), Principal Component Analysis (PCA), and Singular Value Decomposition, are the most common types of unsupervised algorithms. Currently, computers are far from matching human learning abilities; however, in certain practical applications, ML has demonstrated impressive outcomes, even surpassing human capabilities. Within clinical settings, ML technology can aid in lesion localization to a certain extent, leading to a reduction in misdiagnosis rates and an improvement in accuracy. Its application in early breast cancer screening has yielded significant results, particularly when combined with mammography X-ray, ultrasound, breast MRI, and pathological diagnosis, alongside other auxiliary examinations.

DL, as a subset of ML, is capable of automatically extracting meaningful features from big data (14), including the development of image recognition in three stages: text recognition, digital image recognition, and object The basic structure of DL comprises the input layer, hidden layer, and convolutional layer. The hidden layer further consists of the convolutional layer, pooling layer, and fully connected layer (15). The input layer primarily handles data input into the convolutional layer for consolidation purposes, such as feature scaling and data enhancement (16). The primary function of the convolutional layer is to perform feature extraction and calculate the convolution results of data feature mapping using trainable convolutional filters and bias parameters (17). The pooling layer filters and consolidates the features, which are then fed into the fully connected layer for non-linear combination and output (18). The fully connected layer is a structure where every neuron in two adjacent layers is interconnected. Following this layer, the output layer produces the classification result or probability. DL models have extensive applications, encompassing various architectures such as deep neural networks (DNNs), autoencoders (AEs), deep belief networks (DBNs), deep convolutional neural networks (CNNs), recurrent neural networks (RNNs), and generative adversarial networks (GANs).CNN is one of the most representative algorithms in DL, known for its ability to extract high-level information from similar features located at different spatial positions within the input signal (19). Consequently, CNN has achieved significant success in visual recognition and speech recognition tasks (19, 20), particularly excelling in visual recognition due to its high-performance advantages in image processing.

This narrative review provides a comprehensive review of the progress and challenges in the field of ML and DL for mammography, aiming to facilitate researchers’ understanding of the latest advancements. Several published review papers have been presented in the last few years. However, all of them have only been addressed one side focusing on one application or topic, such as Sechopoulos et al. (21) for breast cancer detection with mammography, Gastounioti et al. (22) for breast cancer risk prediction with mammography, Computer-aided breast cancer detection and classification in mammography (23), DL for breast cancer diagnosis (18), DL for Classification of Breast Microcalcifications (24), and etc. This review paper has a distinctive focus compared to previous articles for the following reasons. Firstly, we aim to present an up-to-date examination of the advancements in ML and DL techniques applied to mammography for breast diagnosis. This review serves as a valuable reference for the development of new diagnostic methods. Secondly, we comprehensively discuss the utilization of ML and DL in various stages of mammography image processing, including preprocessing, detection of masses and microcalcifications, as well as segmentation and classification. Furthermore, we address the existing challenges associated with ML and DL approaches, such as limited training data, high implementation costs, and suboptimal accuracy in lesion recognition. Lastly, based on our thorough review, we have derived several significant conclusions that can greatly benefit future research endeavors in the field of medical imaging for breast cancer.

## 2 Methods

**Figure 2:**
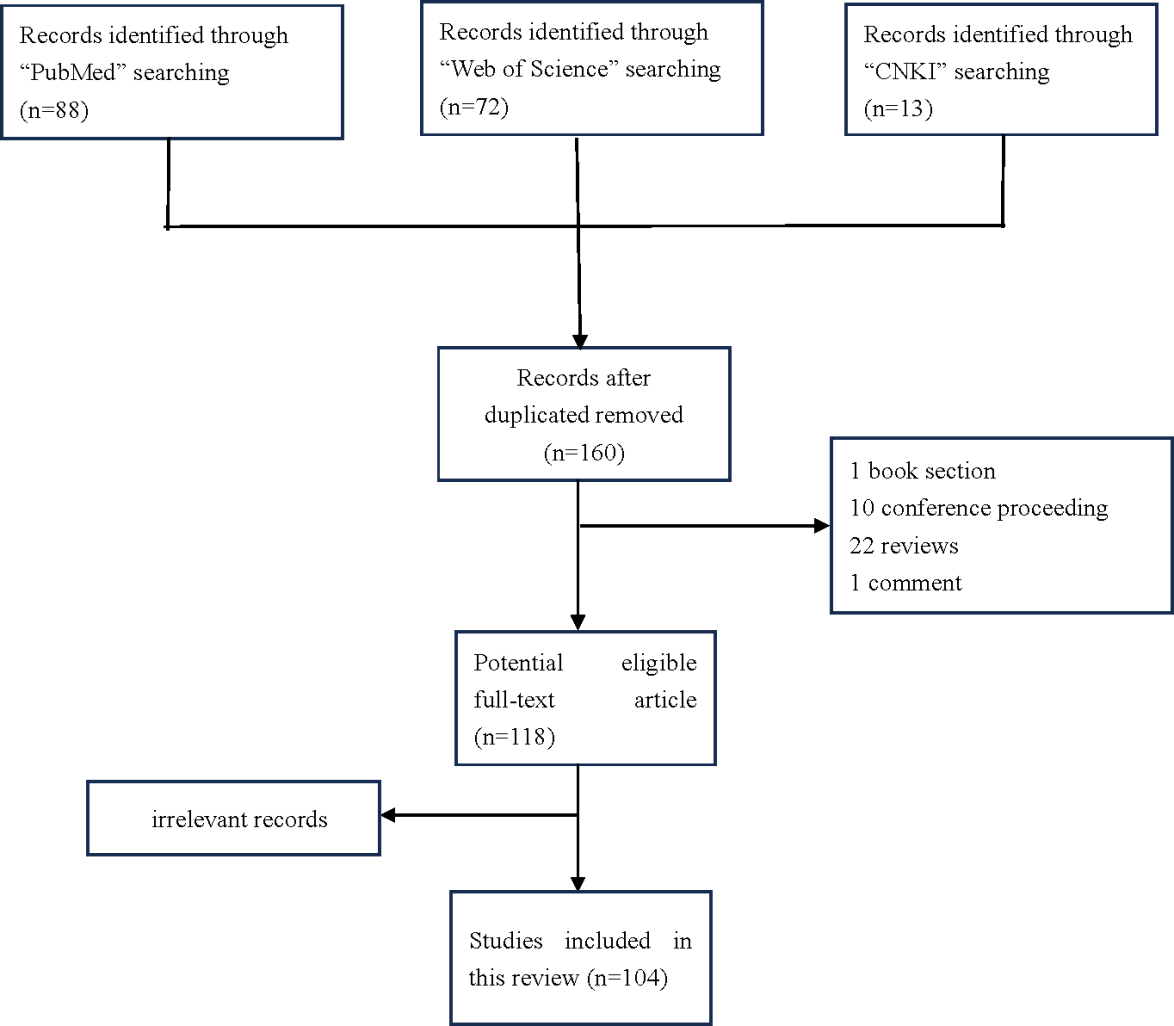
Flow diagram of identification of researches included in the review.

Several databases were searched, including PubMed, Web of Science, and CNKI.The search was limited to studies published between January 2018 and March 2023. Exclusion criteria were applied, which encompassed studies lacking conventional performance metrics such as sensitivity, specificity, area under the receiver operating characteristic curve, and others. Additionally, studies consisting solely of guidelines, review articles, abstracts, animal studies, or with a sample size smaller than 10 were excluded. Following the removal of duplicate studies and those containing redundant or non-novel information, these libraries offer a large number of candidate papers for this study (**Figure 2**).

## 3 Application of machine learning in mammography

**Figure 3:**
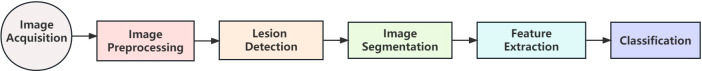
Workflow of computer-aided diagnosis of mammographic images.

As the cause of breast cancer remains unclear, the focus of breast cancer prevention primarily lies in secondary prevention strategies Mammography imaging is a preferred method for clinical breast disease examination due to its simplicity and high recognition rate for early microcalcifications However, mammography has limitations in cases of dense mammary glands, resulting in reduced accuracy (25). ML-based mammograms overcome this limitation by employing ML algorithms to perform tasks such as lesion detection, segmentation, feature extraction, and benign and malignant classification, eliminating the reliance on visual recognition (26) (**Figure 3**).

### 3.1 Preprocessing of mammography images based on traditional machine learning

Image preprocessing is essential due to the challenges encountered in diagnosing mammography images of early breast cancer, including small and irregular calcification points, diverse morphological distribution, poor contrast, and the presence of residual lesion tissues in dense breast tissue. High resolution is often necessary to overcome these challenges, which may require extensive processing time if performed directly on the original images. Common mammography preprocessing methods encompass the removal of background and muscle tissue artifacts, noise reduction, image enhancement, and image resizing. Various algorithms can be applied during the preprocessing stage to improve image quality, including adaptive median filtering, histogram equalization (HE), dynamic thresholding, morphological operations, wavelet transform, Wiener filtering, and contrast-limited adaptive histogram equalization (27). The objective of mammogram preconditioning is to enhance contrast effectively and remove unwanted details within the breast, including the background and pectoral muscles visible in the Medial and Lateral Oblique (MLO) position view, while preserving areas that may hold diagnostic significance (23). Hazarika and Mahant (28)utilized a combination of threshold-based segmentation and morphological manipulation, achieving 98.7% accuracy in identifying breast boundaries and removing the background from the image. Bora et al. (29)employed Hough line transforms and “texture gradient” and “Euclid distance regression” for approximating the chest edge and segmenting muscles from mammograms. Their method successfully removed pectoral muscles from 96.75% of the images. Mammography images often contain speckle noise, which adversely affects image contrast and resolution. Thus, filtering techniques are necessary to remove noise and improve image quality (30). Kavitha et al. (31) proposed the OMLTS-DLCN model, which incorporates adaptive fuzzy median filtering (AFF) as a pretreatment step to remove noise from mammogram images. Arora et al. (32) employed the histogram equalization (HE) method to enhance contrast effectively and improve the image’s edge by expanding its dynamic range as part of the image preprocessing stage.

Mammography image preprocessing primarily aims to remove irrelevant information and noise, while retaining relevant and useful information (33). Continuous advancements in mammography technology have led to significant improvements in picture quality, resulting in reduced noise levels. Due to the similarity between noise and early calcification points, there is a risk of misjudgment during the noise removal process, leading to instances where the noise is not completely eliminated. Given the similarity in grayscale between pectoral muscles and masses, it becomes necessary to normalize the image or enhance contrast to mitigate disturbances caused by the presence of pectoral muscles. Hence, pretreatment plays a vital role in accentuating features, enhancing feature contrast, and improving the reliability of subsequent processing steps.

### 3.2 Lesion detection of mammogram images based on traditional machine learning

Breast cancer originates from the aberrant proliferation of cells in breast tissue, resulting in the development of diverse lesion types, such as asymmetry between the left and right breasts, tissue structure distortion, and the presence of microcalcifications (MCs) and lumps in varying sizes and shapes (34). Breast masses and microcalcifications are prevalent types of lesions encountered in clinical practice. MCs are small calcium deposits commonly found in the breast, appearing as bright spots on mammograms (35). While individual MCs are not highly indicative of breast cancer detection, the formation of microcalcification clusters through the aggregation of microcalcifications serves as an important early sign of breast cancer. Microcalcification clusters, consisting of three or more calcification points per centimeter, can lead to a preliminary diagnosis of early breast cancer. Thus, the detection of microcalcification clusters holds significant value in the early examination of breast cancer. Lumps typically manifest as relatively dense areas (off-white areas) on mammograms. Benign lumps exhibit a round, smooth, and well-circumscribed appearance, while suspicious lumps display irregular, rough, and blurred borders (35).

#### 3.2.1 Microcalcification lesion detection

Duarte et al. (36) demonstrated a technique for segmenting microcalcifications by combining geodesic active profiles with anisotropic texture filtering. The images undergo preprocessing through Alternating Sequential Filtering, and contrast enhancement is achieved using Adaptive Histogram Equalization (CLAHE) technology. The image set utilized in the system is extracted from the DDSM database. This technique achieved an average area overlap measurement of 0.52 ± 0.20, encompassing 87.4% of malignant cases and 86.4% of benign cases. Guo (37) et al. described a system for detecting microcalcified clusters present in digital mammogram images. The zone growth method was employed to eliminate artifacts in mammal X images. The top hat transform and grayscale adjustment methods are utilized for contrast enhancement. The contourlet transform is employed to identify suspicious regions in the breast X image. Calcified clusters are detected using unlinked pulse-coupled neural networks. The proposed system achieved a good accuracy of 95.8%, sensitivity of 96.3%, and specificity of 94.7% when tested on the MIAS and JSMIT databases, respectively. In the (38) study, the researchers proposed a CAD system for mammography microcalcification detection based on a new feature set. They employed statistical observations of classical features (such as higher-order statistics, discrete wavelet transform, and wavelet decomposition) for preprocessing, used the t-test method for evaluation and feature reduction, and achieved good results in sensitivity. Fadil et al. (39) proposed a computer-based automated method for segmenting and classifying breast microcalcifications in mammograms. They utilized discrete wavelet transforms and random forests (DWT-RF) and tested the method on 966 images (322 benign, 322 malignant, and 322 normal). The results showed that DWT-RF achieved a sensitivity of 93%, specificity of 97%, a false positive rate of 3%, and an accuracy of 95%. Moreover, the area under the ROC curve was 0.92, which is comparable to the latest methods and other existing classifiers. Suhail et al. (40) developed a novel method for classifying benign and malignant microcalcifications. They utilized the improved Fisher linear discriminant analysis (LDA) method to perform a linear transformation of segmented microcalcified data and employed junction SVM variants to classify between the two classes. The results demonstrated an average accuracy of 96%.

Recently, the detection of microcalcification points in mammography has gained significant attention as a challenging research area. Despite numerous methods proposed for detecting microcalcification points. However, due to the complex structure of mammography X-ray images, uneven background, and the presence of noise similar to microcalcifications, the detection of microcalcification points still poses certain challenges. Current methods have not yielded satisfactory results, with routine examinations missing 10-30% of cases (41). Hence, researchers should prioritize the development of new technologies to enhance the efficiency and accuracy of detecting microcalcification points in the breast. Additionally, most studies continue to rely on breast image databases from Europe and North America, resulting in a limited number of studies focused on dense mammary X-ray images. Furthermore, there is currently no standardized breast image database suitable for Asian women researchers. Therefore, establishing a standardized breast image database specifically tailored for Asia is an urgent concern.

#### 3.2.2 Mass lesion detection

Huang et al. (42) conducted a retrospective analysis of mammography images, encompassing 124 benign breast masses and 139 malignant breast masses. They extracted the texture features of mammography images and trained four models, namely, linear discriminant analysis (LDA), logistic regression, RF, and SVM, using the training set data. The performance of these models was then verified using the validation set. The RF model exhibited a higher compliance rate, Kappa coefficient, and AUC value in both the training and validation sets. These results highlight the advantages of ML models based on the texture features of mammary X-ray images in distinguishing between benign and malignant breast masses. Tourassi et al. (43) extracted the masses from mammography images and created a region of interest (ROI) database based on the ROI label information of the breast masses in the DDSM database. They employed mutual information as a similarity measure between the template image and the image block to be matched. By calculating the similarity between all suspected mass areas and the template image, they sorted the similarity values and selected the area with the highest matching degree as the detected breast mass. Yu et al. (44) combined median filtering, morphology, and Sobel edge detection to acquire the initial rough edge of the mass. They subsequently employed gradient vector flow snake (GVF-Snake) and gradient map adjustment to perform the final mass segmentation.

Traditional methods for lesion area segmentation can be categorized into region-based, threshold-based, edge-based, feature-based, and theory-based approaches. However, due to the irregular shape of lesions, irregular boundaries, and the presence of grayscale heterogeneity within lesions, relying solely on a single segmentation method often fails to achieve optimal results. Thus, the current trend in research is to synthesize different methods to enhance segmentation accuracy. Additionally, lesion detection methods are frequently combined with segmentation algorithms to simultaneously segment the lesion area and determine the presence of lesions, thereby improving the effectiveness of computer-aided diagnosis.

### 3.3 Segmentation of breast lesion areas based on traditional machine learning

Accurate segmentation of the breast lesion area is fundamental to mammography-assisted diagnosis technology as it serves as the basis for subsequent feature extraction and classification of breast lesions. Generally, the irregularity of a lump’s shape correlates with its malignancy level (45). Traditional methods for breast lump segmentation typically involve area-based algorithms (46), contour-based algorithms (14), threshold segmentation (47), edge detection (**Table 1**). Li et al. (48) developed SAP-cGAN, a mammography mass segmentation model based on an enhanced conditional generative adversarial network (cGAN). The model incorporates a superpixel averaging pool layer in the cGAN decoder, utilizing superpixels to enhance boundary segmentation. Additionally, a multi-scale input strategy is employed to enable the network to learn scale-invariant features and improve robustness. The study demonstrated significant qualitative and quantitative improvements of SAP-cGAN over baseline cGAN and other methods in mammogram mass segmentation using CBIS-DDSM and INbreast datasets. However, the SAP-cGAN model has limitations when applied to mass segmentation involving complex tissue structures. Kozegar et al. (49)propose a two-stage segmentation method that incorporates shape information from training samples. In the first stage, they utilize a novel adaptive region growth algorithm to estimate the mass boundary roughly. Based on the volume and roundness of the training samples, a Gaussian mixture model is employed to determine the algorithm’s similarity threshold. In the second stage, they introduce a novel deformable model based on geometric edges, using the results from the first stage as the initial profile. The study demonstrates the effectiveness of the proposed supervised method in achieving accurate mass segmentation results measured by the Dice coefficient. Alam et al. (50) proposed a novel technique for segmenting microcalcification (MC) clusters by employing a series of morphological operations. The method aims to enhance the accuracy of MC cluster segmentation by selecting the most significant features from the segmented image. These selected features facilitate the generation of the final output in the CADx pipeline. Jen et al. (51)proposed an anomalous feature detection method for mammography based on a novel abnormality detection classification approach. The method utilizes the gray value quantification method to extract five features for detecting the region of interest in the segmented mammogram image. Principal Component Analysis (PCA) is then employed to determine the weight. Experimental results demonstrate that the sensitivity of the method, combined with feature weight adjustment, reaches 88% and 86% on the MIAS dataset and DDSM dataset, respectively. Shi et al. (52) propose an automated image processing pipeline that primarily relies on pixel clustering without training to estimate breast boundaries and simultaneously characterize breast tissue. This pipeline includes skin boundary estimation, breast segmentation, and calcification detection.

**Table 1:** Introduction to lesion segmentation.

Method	Merits	Demerits
Threshold-based segmentation	Simple and easy to operate, high computing efficiency	The complexity of breast images arises from the similarity between high-density tissues and glands in terms of their gray values, making it challenging to identify an appropriate threshold. Consequently, this adversely affects the segmentation of breast masses.
Template-based matching	The extraction of the lump’s area is based on the characteristics of the breast mass and the template’s similarity to the mass.	The calculation of a large number of organizational templates is costly.
Based on a specific model	The method is relatively simple, eliminating the need for complex feature extraction schemes and detailed segmentation of breast tissue.	Strict reliance on the initial contour position is necessary, focusing solely on the grayscale information of the edges in edge understanding.
Combined segmentation	The combination of multiple features enables a more accurate fitting of the characteristics of a breast mass.	The algorithm exhibits complexity and is prone to over-detection.

Lesion segmentation poses significant challenges due to the highly irregular edges of malignant mass lesions, making it difficult to achieve accurate resultsusing a single segmentation method. Additionally, the presence of high-density breast tissue can lead to erroneous segmentation of the dense area as a breast mass. Therefore, addressing these issues necessitates the adoption of a comprehensive range of methods to enhance segmentation accuracy in the future.

### 3.4 Feature extraction and classification of breast lesions based on traditional machine learning

Following pretreatment and accurate segmentation of regions of interest (ROIs) in mammograms, various features can be extracted to classify ROIs as normal, benign, suspect, or malignant microcalcifications (MCs). Common feature extraction methods employed in the extraction stage of mammary lesion features include grayscale co-occurrence matrix (GLCM), multidimensional co-occurrence matrix, independent component analysis, genetic algorithm, two-dimensional PCA, wavelet and curvature methods, and PCA. These methods are utilized to extract features like entropy, skewness, variance, and kurtosis (27).In 2017, Khan et al. (53) employed Gabor filters to extract features from textured micropatterns at various scales and orientations. They utilized Linear Discriminant Analysis (LDA) and Principal Component Analysis (PCA) for dimensionality reduction and employed a weighted SVM based on successive reinforcement learning for classification. The method achieved an average accuracy range of 68% to 100%.Ghazouani et al. (54) propose a fully automated breast cancer diagnostic method that utilizes small training datasets. The method extracts features from mammography images using a genetically programmed descriptor that leverages statistics of local binary pattern-like local distributions defined at each pixel. This approach yields promising results for both content-based retrieval and classification problems. Additionally, The authors (55) present a computer-aided diagnosis (CAD) system for classifying breast masses within mammogram ROIs as malignant or benign. The system employs 13 features based on GLCM to characterize textures. These extracted features are inputted into a SVM classifier, achieving an accuracy of 94%.Vijayarajeswari et al. (56) employed the Canny edge detector, followed by the Hough transform, to extract local texture features. They extracted four types of intensity-based features (mean, entropy, standard deviation, and variance) and utilized them as input for training classifiers. This method achieved an accuracy of 94% in distinguishing normal and abnormal breast tissue. In a study by (57), Haralick’s features were extracted from ROI images, followed by nuclear principal component analysis to reduce the dimensionality of the feature vectors. Subsequently, a wrapper-based parameter optimization kernel extreme learning machine was employed to identify the most significant features from the simplified feature vector.Finally, a wrapper-based parameter optimization kernel extreme learning machine is utilized to select the most prominent features from the simplified feature vector. The multi-level classification accuracy reached 92.61% in the digital breast screening database. Omondiagbe (58)presents a hybrid approach for breast cancer diagnosis that employs Linear Discriminant Analysis (LDA) to reduce the high dimensionality of features and subsequently applies a new dimensionality reduction feature dataset to Support Vector Machines (SVMs). The method achieved an accuracy of 98.82%, sensitivity of 98.41%, specificity of 99.07%, and an area under the receiver operating characteristic curve of 0.9994. SINGH et al. (59) initially enhanced the region of interest using morphological operations. They then extracted cluster shape features and cluster texture features and employed SVM for classification. The feature set was augmented with a set of shape features obtained using the recursive subsampling method, resulting in improved classification accuracy, with an achieved accuracy of 94.25%. Experiments demonstrated that the proposed classification system effectively reduces classification errors and enhances the efficiency of accurate diagnosis. Fanizzi et al. (60) propose an automated binary model for tissue classification in digital mammograms. The model utilizes statistical features obtained through multiscale decomposition of images based on Haar wavelet transforms, as well as points of interest and corners detected using the Speeded Up Robust Feature and Minimum Eigenvalue Algorithm. State-of-the-art machine learning classifiers, such as RF, are trained with these features to address binary discrimination tasks. The proposed model’s performance is evaluated through cross-validation on 260 regions of interest (ROIs). Experimental results demonstrate the model’s excellent prediction performance, with median Area Under the Curve (AUC) values of 98.16% and 92.08% for normal/abnormal and benign/malignant problems, respectively, along with accuracies of 97.31% and 88.46%.Ghasemzadeh et al. (61) extracted feature vectors from mammogram images using the Gabor wavelet transform. They conducted tenfold cross-validation through multiple experiments to analyze the data complexity in each fold. The achieved results showed an average accuracy above 0.939, average sensitivity up to 0.951, and average specificity greater than 0.92. Naseem et al. (62) present an automatic detection system for breast cancer diagnosis and prognosis based on a classifier ensemble. They compare various ensemble models and ML-based test models with and without upsampling techniques on two benchmark datasets. They also investigate the impact of using balanced class weights on prediction datasets and compare the performance with other methods. The results demonstrate the superiority of the ensemble method, achieving an accuracy rate of 98.83%. Dhahbi et al. (63) proposed a grayscale structural analysis method to characterize the region of interest in mammography. They studied the utilization of research methods such as GLCM, fractal analysis, Hilbert image representation, Kolmogorov-Smirnov distance, and maximum subregion descriptors. By extracting features directly from the entire region of interest, the proposed method not only avoids the challenging problem of breast mass segmentation but also considers the texture surrounding the lesion, which significantly aids in breast cancer diagnosis. Additionally, several classifiers including RF, SVM, and decision trees were employed to differentiate between normal tissues and masses. Empirical evaluation using a large database of challenging suspicious regions extracted from the DDSM database demonstrated the effectiveness of the proposed method in reducing false positives in mammography mass detection.

In the clinical diagnosis of breast cancer, it is essential to consider multiple types of lesions, including masses and microcalcifications. Simultaneous classification of benign and malignant lesions across different types is required. Currently, there is a scarcity of research on classification methods for multi-class lesions, and the achieved classification results are also subpar. Addressing this gap in research is a crucial direction for future studies.

## 4 Application of deep learning in mammography

The continuous development and optimization of DL models and algorithms have significantly expanded the application of DL in mammography image analysis (56). **Table 2** presents a compilation of state-of-the-art mammography imaging methods that employ DL techniques

**Table 2:** Literature comparison of different studies on breast cancer.

Reference	Database	Method	Merits	Demerits	Results ACC [%]	Results SEN/SPEC [%]	Results AUC	Results Other
(64)	mini-MIAS	median filter, CLAHE	high accuracy, short runtime	Insufficient sample size	98.98%mini-MIAS)	—	—	—
	INbreast	Deep lab v3+ model.			99.12% (INbreast)			
(65)	DDSM	YOLO detector and InceptionResNet-V2 classifier	fully automated end-to-end deep learning structures do not require user intervention	The size of training and testing annotated breast lesion images are still limited	99.17 (DDSM)	—	—	—
	INbreast				97.27% (INbreast)			
(66)	CBIS-DDSM INbreast	a transfer learning approach	the patch classification is based on the classification of the central pixel.	The study was limited to patch classification, Accuracy is affected by changes in stride values	—	—	—	TPR: 0.98 (CBIS-DDSM)
		CNN models						0.91(INbreast)
(67)	DDSM 、INbreast	an innovative dual-path CNN architecture	automatic segmented mask for mass classification, Independent of resolution	No feature extraction and classification	96.1	85.4/98.1	0.85(DDSM)	—
							0.93(INbreast)	
(48)	CBIS-DDSM	SAP-cGAN	improve breast mass segmentation performance	masses with complex tissue structures are not ideal	—	—	—	CBIS-DDSM:Dice: 93.37%; Jaccard scores:87.57%,
	INbreast							INbreast: Dice91.54% ;Jaccard scores 84.40%
(31)	Mini-MIAS	OMLTS-DLCN: AFF;OKMT-SGO; BPNN	The classification of masses is more effective	No classification	98.50 (Mini-MIAS)	—	—	—
	DDSM				97.55 (DDSM)			
(68)	BCDR-FM	ADL-BCD:	The improvement of the whole process of image processing	There is only one dataset	0.9607	—	—	—
		Gaussian filter;Tsallis entropy; ResNet 34; COA ; WNN						
(69)	Published mammary images	SKMAT-U-Net	Enhance segmentation performance	Feature extraction is not mentioned	0.961	0.981	0.949	
(70)	INbreast;CBIS-DDSM;a private dataset obtained from Cheng Hsin General Hospital in Taiwan	ConnectedSegNets	Image segmentation efficiency is improved	No feature extraction and classification	—	—	—	Dice:
								92.86% (INbreast)
								96.34%(CBIS-DDSM)
								92.25%(private dataset)
(71)	DDSM	the mathematical morphology method; the image template matching method;BD-CNN ; PSO	Increased efficiency in mass identification	No feature extraction and classification	85.82	—	—	—
(72)	CBIS-DDSM	YOLO+LOGO	the improved performance than state-of-the-art models in both breast mass detection and segmentation.	No feature extraction and classification	—	—	—	CBIS-DDSM:F1:74.52、IoU:64.04
	INBreast							INBreast:F1:69.37、IoU:61.09
(73)	an advanced medical center for preventive medicine established by Osaka City University Hospital.	SR+CNN	improve the visibility of microcalcifications	The recognition of non-calcified lesions is slightly poor	—	—	—	—
(74)	U-Net	DDSM	Improve the accuracy of lump detection	Lesion testing for dense breasts may be false-positive	85.95	92.32/80.47	86.4	—
(75)	CNN	DDSM	extract deep features	limited by the amount of data.	94.92(Normal/Abnormal)	SEN:	94.72(Normal/Abnormal)	—
					95.24(Benign/Malignancy)	96.52(Normal/Abnormal)	95.01(Benign/Malignancy)	
						96.11(Benign/Malignancy)		
(76)	SRL+MCMTL	CBIS-DDSM	Good breast mass detection	No classification	—	—	—	0.812 TPR@2.53 FPI (CBIS-DDSM)
		INBreast						0.919 TPR@0.12 FPI(INBreast)
(77)	multi-scale residual networks and densely connected networks;CBAM	DDSM	The extraction effect of mass lesions is good	Microcalcified lesions are poorly detected	97.19	Sen:97.19	95.76	—
(15)	ResNet 18; transfer learning	BreakHis	The accuracy of classification of benign and malignant lesions is improved	Only one dataset	97.81–99.70	—	—	—
(78)	ResNet50V2;ResNet101V2;ResNet152V2	CBIS-DDMS;INbreast;a private dataset	Better classification performance	No feature extraction	No feature	85.38(CBIS-DDMS)	—	0.94(CBIS-DDMS)
					Segmentation.and extraction	99 (INbreast)		1.0 (INbreast))

— indicates that the article is not mentioned.

### 4.1 Deep learning-based detection of mammogram lesions

Leong et al. (79) proposed an adaptive transfer learning deep convolutional neural network for segmenting mammogram images with calcified cases, aiming to assist in early breast cancer diagnosis and intervention. Honjo et al. (73) p proposed a deep-learning-based super-resolution (SR) model based on DL for identifying microcalcifications (MCs) in mammography images. Researchers conducted visual and quantitative comparisons between pre-processed and post-processed images, demonstrating the model’s potential in detecting and diagnosing microcalcifications. Zeiser et al. (74) introduced a U-Net-based model for diagnosing CAD systems in digitized mammograms, enabling lesion monitoring over time. The proposed methodology involves (1): Preprocessing, including removal of irrelevant information, contrast enhancement, and area of interest extraction (2); Data enrichment through horizontal mirroring, scaling, and resizing of images (3); Training based on a six-membered U-Net model with diverse characteristics. The results indicated that the best model achieved a sensitivity of 92.32%, specificity of 80.47%, accuracy of 85.95%, Dice index of 79.39%, and AUC of 86.40%.Al-atari et al. (65) presented an integrated CAD system for breast lesion detection and classification using DL. They first utilized the YOLO detector based on DL to evaluate breast lesion detection in the DDSM and INBREAM mammogram databases, achieving overall detection rates of 99.17% and 97.27%, respectively. Using the detected breast lesions, the average overall accuracy of the CNN, ResNet-50, and InceptionResNet-V2 classification models for the DDSM and INbreasts datasets was reported as 94.50%, 95.83%, 97.50%, and 88.74%, 92.55%, 95.32%, respectively. The DL-based YOLO detector improves lesion detection accuracy in mammography X-rays, thereby enhancing the classification model’s diagnostic performance for breast lesions. Sun et al. (71) proposed a novel breast mass detection method that integrates mathematical morphology, image template matching, CNN-based breast mass detection, and a breast mass bounding box regression model using the particle swarm algorithm. The proposed method’s detection performance was experimentally evaluated on the mammography image dataset DDSM and compared with the state-of-the-art breast mass detection method. Niu et al. (77) utilized a convolutional neural network method to classify benign and malignant masses in mammography films. They employed a multi-scale residual network and a dense connectivity network as the backbone network to extract features from global and local image patches. Additionally, they employed the Convolutional Block Attention Module to enhance the feature expression ability of the network. Finally, the characteristics of multi-scale image patches were fused to achieve the classification of benign and malignant breast masses.

### 4.2 Deep learning-based segmentation of breast lesions

Su et al. (72) eveloped a deep-learning model architecture for detecting and segmenting breast cancer masses using mammography. The model combines YOLO (You Only Look Once) and LOGO (Local-Global) architectures for quality inspection and segmentation. Firstly, YoloV5L6 was employed to locate and crop the breast lump in the mammogram. Secondly, to achieve a balance between training efficiency and segmentation performance, the researchers modified the LOGO training strategy by training the entire image and cropping the image on the global and local Transformer model branches, respectively. These two branches are then merged to make the final split decision. The results demonstrate that the proposed YOLO-LOGO model exhibits higher efficiency, improved performance, reduced computing requirements, and enhanced versatility and accuracy in computer-assisted breast cancer diagnosis. Zhou et al. (64) present a novel DL-based method for extracting breast regions that combines various pretreatment techniques, including noise suppression using median filters, contrast enhancement using CLAHE, and semantic segmentation using Deep lab v3+ models. The method is trained and evaluated on the mini-MIAS dataset and also evaluated on the INbreasts dataset. The results surpass those of recent studies, highlighting the model’s capability to maintain accuracy and runtime advantages across different databases with varying image resolutions.Li et al. (67) propose a novel DL framework for processing mammogram images that involves mass segmentation and simultaneous prediction of diagnostic outcomes. Firstly, they construct a quality and context texture learner, known as the Locality Preserving Learner, using a stack of convolutional blocks to map regions of interest to class labels at a relatively large scale. Secondly, they employ the Conditional Graph Learner, which combines graph and CNN, to learn correlations in relatively small-scale ROIs and utilize the extracted segmentation features to enhance the final quality classification performance. The DUAL CORENET framework achieves optimal mammogram segmentation and classification, exhibiting superior segmentation performance at both low and high resolutions. Li et al. (48) propose a novel network architecture for segmenting massive images in digital mammograms. The architecture combines two modules within the main basic cGAN framework: a superpixel average pooling layer and a multiscale input module. These modules provide prior boundary information and scale-invariant features. The model’s performance is evaluated on large-scale images from two commonly used datasets, CBIS-DDSM and INBREST. The model achieves impressive results in terms of Dice and Jaccard scores, accuracy, specificity, and sensitivity. For the CBIS-DDSM dataset, the Dice and Jaccard scores are 93.37% and 87.57%, respectively. For the INBREASTS dataset, the Dice and Jaccard scores are 91.54% and 84.40%, respectively. These findings indicate that the proposed model outperforms current state-of-the-art breast mass segmentation methods. Chakravarthy et al. (69) introduce an enhanced version of U-Net called SKMAT-U-Net. This model incorporates a selective kernel with an attention mechanism to adaptively adjust the network’s receptive fields. It combines feature maps extracted through extended and standard convolution operations. The researchers then integrate four attention loss functions based on the traditional cross-entropy loss function to form a U-Net using the Mixed Attention Loss Function. The proposed model effectively segments lesions in ultrasound images of the mammary glands. Alkhaleefah et al. (70) developed a DL model called Connected SegNets for segmenting breast tumors from X-ray images. In the proposed model, two SegNet architectures are connected by skip-the-loop connections between their layers. To enhance the model’s robustness to noise during training, the original SegNet’s cross-entropy loss function is replaced by the intersection-over-union (IoU) loss function. Contrast Limited Adaptive Histogram Equalization (CLAHE) is applied to all datasets to enhance the compressed area and smooth pixel distribution. Additionally, two image enhancements, rotation and anti-warping, are employed to augment the training data and mitigate overfitting. Experimental results demonstrate the superiority of the proposed connected segmented network model over existing methods, as evidenced by higher Dice score and IoU score. Rodriguez-Ruiz et al. (80) proposed an automatic pectoral muscle segmentation model based on the U-Net DL architecture. The model was trained using 136 DBT images acquired by a single system. It was then evaluated on 125 images of three different types: Digital breast tomosynthesis (DBT), synthetic mammographic image (SM), and digital mammography (DM). The obtained Dice Similarity Coefficients (DSCs) ranged from 0.947 to 0.970, a visually determined range ensuring adequate segmentation.

### 4.3 Feature extraction and classification of breast lesions based on deep learning

Zhang et al. (75) proposed a multi-view feature fusion network model for classifying mammograms using a multi-scale attention-dense network as the backbone network for feature extraction. The model incorporates two CNN branches to extract features from mammography images captured from different perspectives, enabling the network to leverage a broader range of spatial information. Additionally, a multi-scale convolution module is introduced to extract features at various scales within the images. Experimental results demonstrate the model’s strong performance in both classification tasks. The model achieves an accuracy of 94.92% and 95.24%, sensitivity of 96.52% and 96.11%, and AUC values of 94.72% and 95.03% for classifying normal and abnormal mammograms, and benign and malignant mammograms, respectively. Shen et al. (76) propose a novel DL framework composed of two primary stages: Suspicious Region Localization (SRL) and Multicontext Multitask Learning (MCMTL). In the first stage, SRL is responsible for generating the region of interest (ROI) and extracting multi-sized patches from these suspicious regions. In the second stage, the MCMTL network combines the features of the multi-sized patches from the suspicious areas to perform simultaneous classification and segmentation. The proposed method demonstrates performance that is on par with the most advanced methods reported in the literature. Aljuaid et al. (15) propose a computer-aided diagnostic method for breast cancer image classification utilizing deep neural networks (ResNet 18, ShuffleNet, and Inception-V3Net) and transfer learning. The method leverages BrakeHis’ publicly available breast cancer images and considers various image magnification factors and data augmentation techniques to enhance the classification process. Three deep neural networks were employed to classify the breast cancer images using an image-based approach. The results indicate that the average accuracy for binary and multiclass classification ranged from 97.81% to 99.70%. The researchers concluded that ResNet was the most accurate and efficient classifier among the three models. Baccouche et al. (78) developed a stacked ensemble of ResNet models (ResNet50V2, ResNet101V2, and ResNet152V2) to classify breast masses as malignant or benign and assess their BI-RADS category on a scale of 2 to 6, considering their shape (oval, round, lobulated, or irregular). The results of the proposed method demonstrate improved classification performance compared to individual models and other methods applied to existing benchmark datasets. Agarwal et al. (66) propose a patch-based CNN method for automatic detection of breast lesions in full-field digital mammograms (FFDM). They employ transfer learning by training CNNs on a large public database of digitized mammograms (CBIS-DDSM dataset) and transferring the model to a smaller digital mammogram database (INbreast dataset) for evaluation. VGG16, ResNet50, and InceptionV3 are used as depth feature extractors, and the InceptionV3-based model achieves the best detection results with a true positivity rate (TPR) of 98%. LKavitha et al. (31) proposed the Optimal Multi-Level Thresholding-based Segmentation with DL enabled Capsule Network (OMLTS-DLCN), a breast cancer diagnostic model based on digital mammograms. The model incorporates Adaptive Fourier Filtering (AFF) as a pretreatment step to remove noise from mammogram images. It employs the Optimal Kapur’s based Multilevel Thresholding with Shell Game Optimization (OKMT-SGO) algorithm for segmentation and lesion detection in mammogram images. Additionally, the model utilizes a Capsule Network-based feature extraction method and a Back-Propagation (BP) neural network for breast cancer detection. The diagnostic performance of the OMLTS-DLCN model was evaluated using the benchmark Mini-MIAS dataset and DDSM dataset, achieving high accuracy rates of 98.50% and 97.55%, respectively. Escorcia-Gutierrez (68) proposes an automated DL-based breast cancer diagnosis method called ADL-BCD technology, which employs digital mammograms for the detection of breast cancer. ADL-BCD technology encompasses GF-based preprocessing, Tsallis entropy-based segmentation, ResNet34-based feature extraction, chimp optimization algorithm (COA)-based parameter tuning, and wavelet neural network-based classification. The use of COA-based hyperparameter optimization significantly enhances diagnostic efficiency. The ADL-BCD method was evaluated using a benchmark dataset, and the simulation results demonstrate its superior performance compared to existing methods across various evaluation measures.

### 4.4 Conclusion based on deep learning

The rapid development of DL and the significant improvement in computer performance have made DL a prominent research area for analyzing and processing medical images to aid in lesion diagnosis. Traditional computer-aided diagnosis (CAD) systems lack deep networks and can only extract shallow features, resulting in poor system performance. DL, on the other hand, can automatically extract informative features from medical images, including those that may not be easily visible to the naked eye. This capability greatly enhances the diagnostic accuracy and efficiency, particularly in the context of breast lesion diagnosis. Recent studies have demonstrated that DL applied to breast cancer screening is approaching the diagnostic proficiency of experienced radiologists (81).. However, the challenge of relying on ROI annotation in DL techniques has yet to be effectively addressed. Therefore, further research should be conducted to explore DL techniques that reduce the dependency on ROI annotation. However, the challenge of relying on ROI annotation in DL techniques has yet to be effectively addressed. Therefore, further research should be conducted to explore DL techniques that reduce the dependency on ROI annotation. Some researchers (82, 83) have employed various DL techniques to detect and classify suspicious areas in mammography images, resulting in improved model performance to some extent. Moreover, considering that DL training and validation sets require extensive data, the creation of a large publicly available dataset with high accuracy, resolution, and diversity becomes necessary. This dataset will facilitate the training of CAD models with superior performance. Consequently, the integration of DL into mammography CAD systems represents a promising direction for advancing computer-aided diagnosis technology for mammography images.

## 5 Discussion

Mammography is a widely used method for early diagnosis of breast cancer. It offers high-resolution X-ray images that enable the visualization of different layers of breast tissue. This technique is effective in detecting breast hyperplasia, benign and malignant tumors, as well as disorders in breast tissue structure (84). Additionally, mammography provides clear images that facilitate before-and-after comparisons, making it highly significant for early detection, diagnosis, and treatment. This narrative review provides an analysis and discussion on the current state of computed breast diagnosis technology, encompassing both traditional ML methods and DL. Firstly, the paper introduces the definition and fundamental theoretical knowledge of ML and DL. It then explores the research on the application of traditional ML techniques in mammography, including preprocessing, feature extraction, lesion segmentation, and benign and malignant classification. Subsequently, the paper delves into the application of DL in mammography, covering lesion detection, segmentation, and classification, while briefly outlining the advantages and disadvantages of DL. Despite the deepening of research on computer diagnosis of breast cancer, there are still many challenges:

(1) The available sample data for glandular structure distortion and asymmetric dense shadow is limited and lacks standardization. Currently, most research focuses on mass and microcalcification detection, neglecting the investigation of glandular structure distortion and asymmetric dense effects. Therefore, it is crucial for researchers to prioritize the development of models for detecting multiple lesions and creating automated tools for identifying glandular structure distortion and asymmetric dense effects.
(2) The variability in shapes and edges of suspicious lesions, along with the blurred boundary between the lesion and the surrounding tissues, results in unstable lesion depiction and increases the risk of misdiagnosis. Although ML-assisted breast diagnosis has significantly improved accuracy, establishing a stable lesion identification system and implementing it on a large scale remain challenging research areas. Therefore, there is a need for further improvements in techniques for automated detection and segmentation of breast lesions in mammograms.
(3) Despite the increasing research on DL, its application still faces limitations. Firstly, training a new DL system requires a large amount of raw data. Secondly, the lack of a unified standard in datasets due to technological, equipment, and operator limitations hinders the reproducibility of research findings across different datasets. Moreover, DL technology is costly to learn, and its internal workings are highly complex (85). Furthermore, DL models typically provide output results without easy-to-understand explanations.
(4) There is a lack of data with complete annotations, and the existing database is small. Both traditional ML and DL require a significant amount of labeled data for training. However, obtaining such data is challenging due to the highly specialized and fragmented nature of medical data. Consequently, in future research, it is imperative to expand the dataset and explore the utilization of DL and other approaches to enhance data availability.
(5) Exploring the integration of artificially defined features with deep features to enhance the performance of DL models is a pertinent research question. The conventional approach of feature extraction faces challenges, such as low efficiency, and the high data processing cost associated with DL.

## 6 Conclusion

Breast cancer poses a significant threat to women’s health and mortality, emphasizing the importance of early detection and treatment. Mammograms serve as a highly effective and reliable tool for timely identification and diagnosis of breast cancer (86). CAD systems built on mammography have aided doctors in decision-making and reduced diagnostic errors to some extent, traditional ML-based CAD systems encounter challenges in terms of limited generalization ability, inadequate automation, continued reliance on manual intervention, and a high demand for operators possessing specialized domain knowledge and engineering skills. However, with the advancement of DL research, DL-based CAD systems emerge as a viable solution to address these issues effectively, significantly enhancing the efficiency of breast cancer diagnosis.

## Author contributions

YG: Data analysis and writing original draft, writing. JL and YZ review and editing. RL funding acquisition, methodology, project administration, resources, supervision. All authors contributed to the article and approved the submitted version.
